# Serum IFN-γ levels predict the therapeutic effect of mesenchymal stem cell transplantation in active rheumatoid arthritis

**DOI:** 10.1186/s12967-018-1541-4

**Published:** 2018-06-15

**Authors:** Yi Yang, Xiao He, Rongseng Zhao, Wei Guo, Ming Zhu, Wei Xing, Dongpo Jiang, Chongyang Liu, Xiang Xu

**Affiliations:** 1 First Department, State Key Laboratory of Trauma, Burn and Combined Injury, Daping Hospital and Research Institute of Surgery, Third Military Medical University, Chongqing, 400042 People’s Republic of China; 2 Department of Rheumatology and Clinical Immunology, Daping Hospital and Research Institute of Surgery, Third Military Medical University, Chongqing, 400042 People’s Republic of China; 3 Department of Critical Care Medicine, Daping Hospital and Research Institute of Surgery, Third Military Medical University, Chongqing, 400042 People’s Republic of China

**Keywords:** Mesenchymal stem cell, Rheumatoid arthritis, IFN-γ, Clinical trial

## Abstract

**Background:**

To explore the mechanism of the different clinical efficacies of mesenchymal stem cell transplantation (MSCT) and identify a possible serum biomarker for predicting the therapeutic effect of MSCT in rheumatoid arthritis (RA) patients.

**Methods:**

A total of 105 patients with persistently active RA and poor responses to traditional medication were randomly divided into MSCT and control groups. Outcomes were evaluated according to the 28-joint Disease Activity Score and Health Assessment Questionnaire, serological indicators, regulatory T cell (Treg) to T helper 17 (Th17) cell ratio, and inflammatory cytokine levels. Twelve weeks after MSCT, the outcomes of the MSCT group were evaluated according to the European League against Rheumatism response criteria. Patients with a good or moderate response were added to the response group, and those with no response were added to the no-response group.

**Results:**

No serious adverse events were reported for either MSCT subgroup (28 in the response group and 24 in the no-response group). The therapeutic effects lasted for 48 weeks without continuous administration. Notably, a transient increase in serum IFN-γ (>2 pg/ml) levels was observed in the response group, but not in the no-response group. Furthermore, an increase in IL-10 levels and the Treg/Th17 ratio and a reduction in IL-6 levels appeared 2–3 weeks after the transient IFN-γ increase.

**Conclusions:**

Allogeneic MSCT is safe and feasible, and we propose high serum IFN-γ levels as a potent biomarker for predicting MSCT response.

*Trial registration* chictr.org, ChiCTR-ONC-16008770. Registered 3 July 2016, http://www.chictr.org.cn/showproj.aspx?proj=14820

**Electronic supplementary material:**

The online version of this article (10.1186/s12967-018-1541-4) contains supplementary material, which is available to authorized users.

## Background

Rheumatoid arthritis (RA) is chronic synovitis characterized by articular inflammation, synovial joint damage and deteriorating disability over time. The basic pathogenic mechanisms that initiate and promote RA progression are likely to be systemic and synovial inflammation due to unbalanced immune homeostasis, possibly involving genetic and environmental factors.

In the BeSt study, 508 RA patients were treated with hormones, disease-modifying antirheumatic drugs (DMARDs), or biological agents and were followed for up to 5 years; according to the results, more than 50% of the RA patients did not achieve clinical remission after treatment [1]. Therefore, despite great medical advances, the following problems remain: (i) some patients cannot tolerate DMARDs or biologics; and (ii) biologic agents are expensive and weaken the immune system, thus potentially increasing the risk of infections. Consequently, novel treatment options for RA patients are urgently needed.

Mesenchymal stem cells (MSCs) are multipotent cells derived from bone marrow or other connective tissues, such as the umbilical cord; MSCs have the capacity to self-renew and differentiate into mesenchymal tissues [2]. In addition to their differentiation ability, MSCs have immunomodulatory effects on many diseases [3, 4]. MSCs can inhibit the proliferation of activated peripheral blood mononuclear cells (PBMCs) and T lymphocytes [5] to induce regulatory T cell (Treg) differentiation and inhibit T helper 17 (Th17) cell function [6]; it is through these mechanisms that MSCs exert their immunomodulatory effects on RA. The immunomodulatory abilities of MSCs are thought to depend on interferon-γ (IFN-γ)-induced indoleamine 2,3-dioxygenase (IDO). The unique immunomodulatory properties of MSCs make them a promising candidate cell therapy for tissue repair, potentially for use in treating immune disorders, such as graft-versus-host disease (GVHD) [7], and autoimmune diseases, such as systemic lupus erythematosus (SLE) [8]; however, contradictory results have been reported for using MSC transplantation (MSCT) to treat RA [9, 10]. There are huge individual variations in the clinical efficacy of MSCT for the same disease, but the reasons for this are unclear. For the widespread clinical application of MSCT, a better understanding of the biological properties of MSCs is required. This information would help clarify the mechanisms of MSC-based transplantation for immunomodulation and improve clinical efficacy.

In this study, we aimed to determine the different clinical efficacy of MSCT in RA patients and to identify a possible serum biomarker for predicting the therapeutic effects of MSCT.

## Methods

### Patient eligibility

All patients met the American College of Rheumatology 1987 (ACR1987) criteria for RA classification and had no other autoimmune or systemic diseases. Written informed consent was provided in accordance with the Declaration of Helsinki. The study was registered at Chictr.org (identifier: ChiCTR-ONC-16008770) and approved by the Ethics Committee of Daping Hospital, Third Military Medical University of the Chinese People’s Liberation Army (YIYANLUNSHEN (2016) NO. 007). The enrolled RA patients responded poorly to regular clinical strategies, including DMARDs, non-steroidal anti-inflammatory drugs (NSAIDs), steroids and biologics, or could not tolerate their serious side effects; thus, the patients maintained active disease conditions (Additional file 1: Table S1). Overall, 105 RA patients were enrolled from July 2016 to March 2017.

### Treatment protocol

Patients were randomly divided into MSCT and control groups. Source and preparation of umbilical cord-derived human MSCs (UCMSCs) were detailed in Additional file 2. The patients received either 1 × 10^6^ cells/kg body weight in 50 mL of 1% albumin in physiological saline as the treatment or 50 mL of 1% albumin in physiological saline without UCMSCs as the control via intravenous infusion. If the status of the patient continued to improve, a withdrawal schedule was used to taper off conventional drug treatment in the following order: prednisone acetate, then NSAIDs and DMARDs. All treatment modifications were agreed upon by the rheumatologist in charge.

### Assessment of disease status

**Fig. 1:**
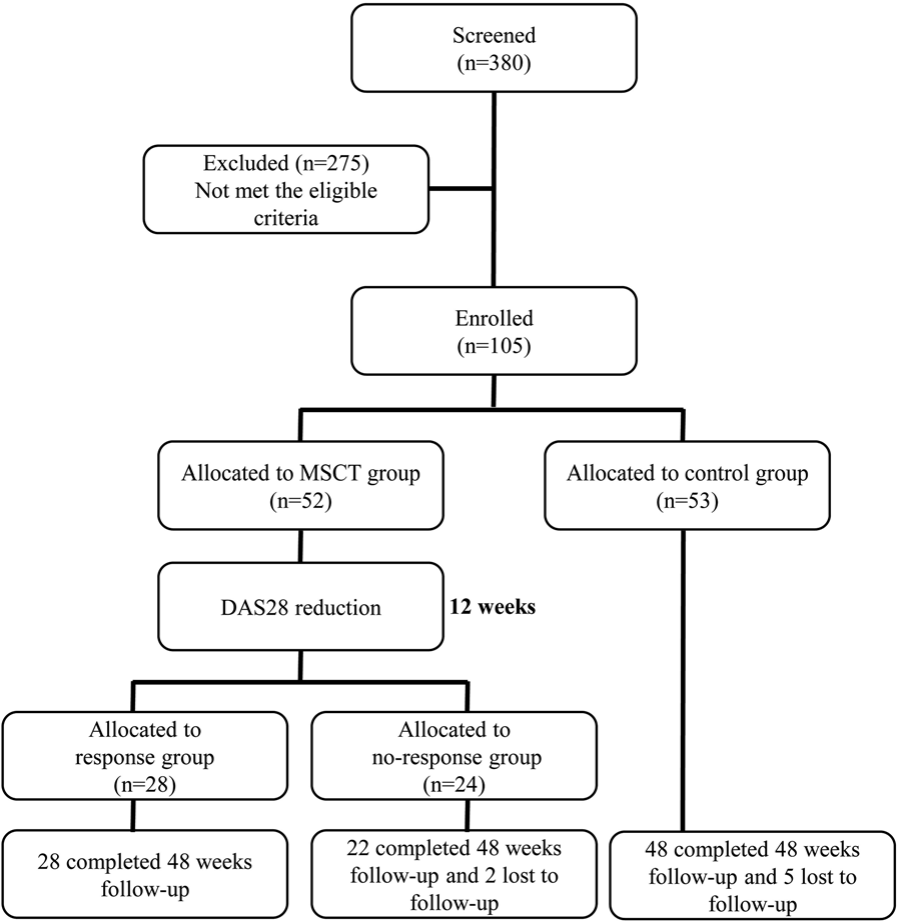
Trail profile. 105 rheumatoid arthritis (RA) patients were enrolled and randomized allocated into two groups. 12 weeks after MSCT, outcomes of the MSCT group patients were evaluated according to the EULAR response criteria, taking into consideration of both current DAS28 value and its reduction after treatment, those with good or moderate response were put into the response group (n = 28), and those with no response were put into the no-response group (n = 24). Most enrolled patients completed 48 weeks follow-up, but there are 2 of the no-response group and 5 of the control group lost to follow-up. MSCT, mesenchymal stem cell transplantation. DAS28, the 28-joint disease activity score

All patients returned for scheduled follow-up visits at 1, 2, 3, 4, 12, 24, and 48 weeks. At these time points, the adverse events, general physical status, serological indicators, and Treg to Th17 cell ratio of the patients were examined and recorded. The 28-joint Disease Activity Score (DAS28) and Health Assessment Questionnaire (HAQ) were assessed at baseline and each follow-up visit for each patient. Specifically, to determine the different clinical efficacies of MSCT in RA patients, outcomes for the MSCT group were evaluated according to the European League against Rheumatism (EULAR) response criteria, which consider both the current DAS28 value and any reduction after treatment. Patients with a good or moderate response were added to the response group, and those with no response were added to the no-response group at 12 weeks after MSCT (Fig. 1).

### Laboratory tests

Serum levels of anti-cyclic citrullinated peptide antibody (anti-CCP) and rheumatoid factor (RF) were determined by indirect immunofluorescence using kits purchased from Euroimmun (Lübeck, Germany). The percentages of peripheral blood Tregs and Th17 cells were analyzed by flow cytometry (NovoCyte™, ACEA Biosciences, San Diego, CA USA). Monoclonal antibodies against CD4, CD25, Foxp3 and IL-17A were purchased from BD. Flow cytometry data were analyzed with NovoExpress software (ACEA Biosciences, San Diego, CA USA). A bead-based multiplex cytokine assay was custom-designed to quantify the following cytokines: TNF-a, IFN-γ, IL-1β, IL-2R, IL-6, IL-8, and IL-10. The assays were performed according to their instructions, and measurements were made with a Luminex 200 system (Millipore Corporation, Bedford, Massachusetts USA).

### Statistical analysis

Statistical analyses were conducted using the *t* test for parametric data and the Mann–Whitney *U* test for non-parametric data. One-way analysis of variance (ANOVA), followed by the Bonferroni test, was used when there were more than two groups. All statistical tests were two-sided, and the significance level was set at P < 0.05. All analyses were conducted with SPSS 17.0 (SPSS, Inc). The data are shown as the mean ± standard error of the mean.

## Results

### Safety evaluation

No serious acute adverse events occurred during or after MSCT. Three patients had chills or fever (≤ 39 °C) after MSC infusion but recovered within 3 h without any intervention. No patients developed graft-versus-host disease (GVHD), and no serious infections occurred. No significant abnormalities were found according to routine blood tests, liver and kidney function analysis, chest radiography, urine analysis, or electrocardiography.

After 48 weeks, the response group (n = 28) showed significant increases in hemoglobin and albumin levels and decreases in platelet levels; these findings indicate immune function improvements (Table 1).

**Table 1:** Safety evaluation on patients between response and no-response group

Measures (normal value range)	No-response		Response	
	Before treatment	After treatment	Before treatment	After treatment
Total protein (65–85 g/L)	73.24 ± 4.32	72.52 ± 3.46	72.63 ± 5.21	74.64 ± 3.68
Albumin (40–55 g/L)	33.23 ± 5.31	32.57 ± 3.54	32.54 ± 4.63	40.63 ± 3.25*
Globulin (20–40 g/L)	30.62 ± 3.57	31.39 ± 4.36	31.58 ± 4.23	32.13 ± 3.86
Cholesterol (3.1–5.72 mmol/L)	4.03 ± 1.34	4.23 ± 1.53	4.15 ± 1.26	4.28 ± 1.93
Triglyceride (0.30–1.7 mmol/L)	1.46 ± 0.69	1.49 ± 0.83	1.51 ± 0.38	1.43 ± 0.58
Creatinine (41–73 μmol/L)	46.53 ± 13.52	45.36 ± 12.38	43.36 ± 12.78	45.25 ± 14.21
Urea (2.6–7.5 mmol/L)	4.23 ± 1.35	4.34 ± 1.28	4.12 ± 1.24	4.15 ± 1.87
Fasting blood glucose (3.9–6.1 mmol/L)	4.74 ± 0.83	4.84 ± 0.96	4.67 ± 0.73	4.75 ± 0.86
White blood cell (3.5–9.5) × 10^9^	5.65 ± 1.35	5.78 ± 1.26	5.84 ± 1.39	5.59 ± 1.47
Hemoglobin (115–150 g/L)	102.35 ± 18.79	103.24 ± 19.21	101.15 ± 19.82	113.46 ± 16.62*
Platelet (94–268) × 10^9^	253.23 ± 68.31	262.31 ± 72.78	263.12 ± 70.24	223.19 ± 65.87*

No-response group: n = 22; Response group: n = 28

Value: mean ± SEM, *t* test, * P < 0.05

### Assessment of disease activity

**Fig. 2:**
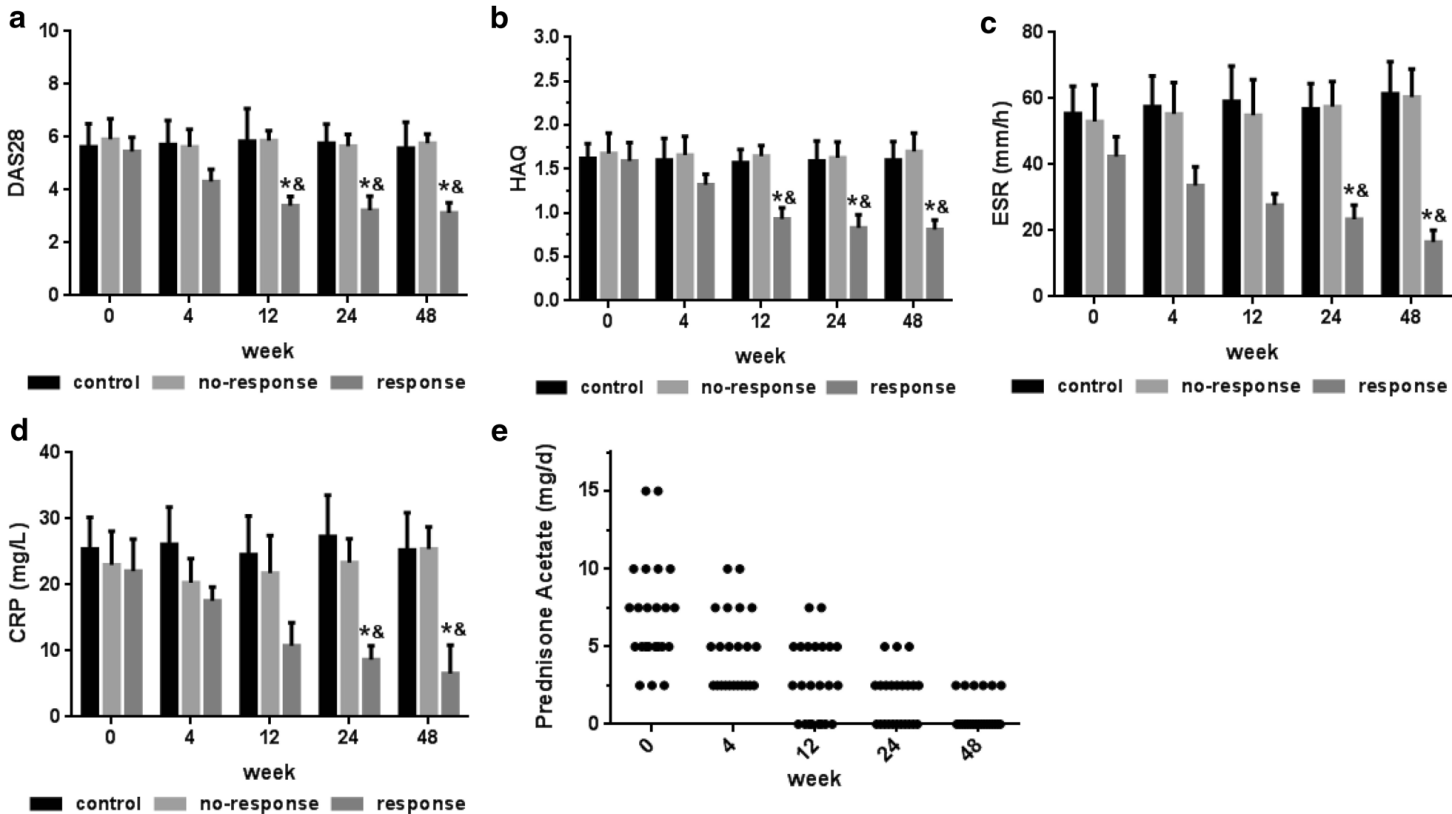
Assessment of disease activity. DAS28 value, HAQ score, ESR and CRP level before and after MSCT for the groups. **a** DAS28 value; **b** HAQ score; **c** serum ESR; **d** serum CRP level; **e** changes in prednisone acetate dosage after MSCT in the response group; each dot represents one patient in the response group (n = 23). *P < 0.05, versus the response group before MSCT; ^&^P < 0.05, versus the no-response and control groups according to one-way ANOVA. The error bars indicate the SEM. *CRP* C-reactive protein, *ESR* erythrocyte sedimentation rate

During the 12 weeks of follow-up after MSCT, 28 patients in the MSCT group had rapidly improved clinical symptoms with decreases in disease activity and drug dosage after MSCT. However, the other 24 patients in the MSCT group and the patients in the control group (n = 53) did not show signs of improvement. According to the study protocol, we divided the patients in the MSCT group into a response group (n = 28) that had a good or moderate response and a no-response group (n = 24) (Fig. 1) according to the EULAR response criteria, which are based on the DAS28 [11]; no significant differences among the groups were detected at baseline (Additional file 1: Table S1). In agreement with the decrease in C-reactive protein (CRP) levels and the erythrocyte sedimentation rate (ESR), the HAQ and DAS28 values of the response group were significantly decreased 12 weeks after MSCT (Fig. 2). These findings indicated an improvement in the disease status. In addition, most of the patients in the response group maintained these therapeutic effects for 48 weeks without continuous administration. However, 2 (8%) experienced relapse, which was indicated by an increase in the ESR and CRP levels and joint swelling and pain at 24 weeks. What is more, the prednisone acetate doses were successfully reduced stepwise in 23 patients in the response group after MSCT (Fig. 2e) and their drug plans were not modified 6 months before enrollment. Most enrolled patients completed 48 weeks follow-up, but there are 2 of the no-response group and 5 of the control group lost to follow-up.

### Serological autoantibody profile findings

**Fig. 3:**
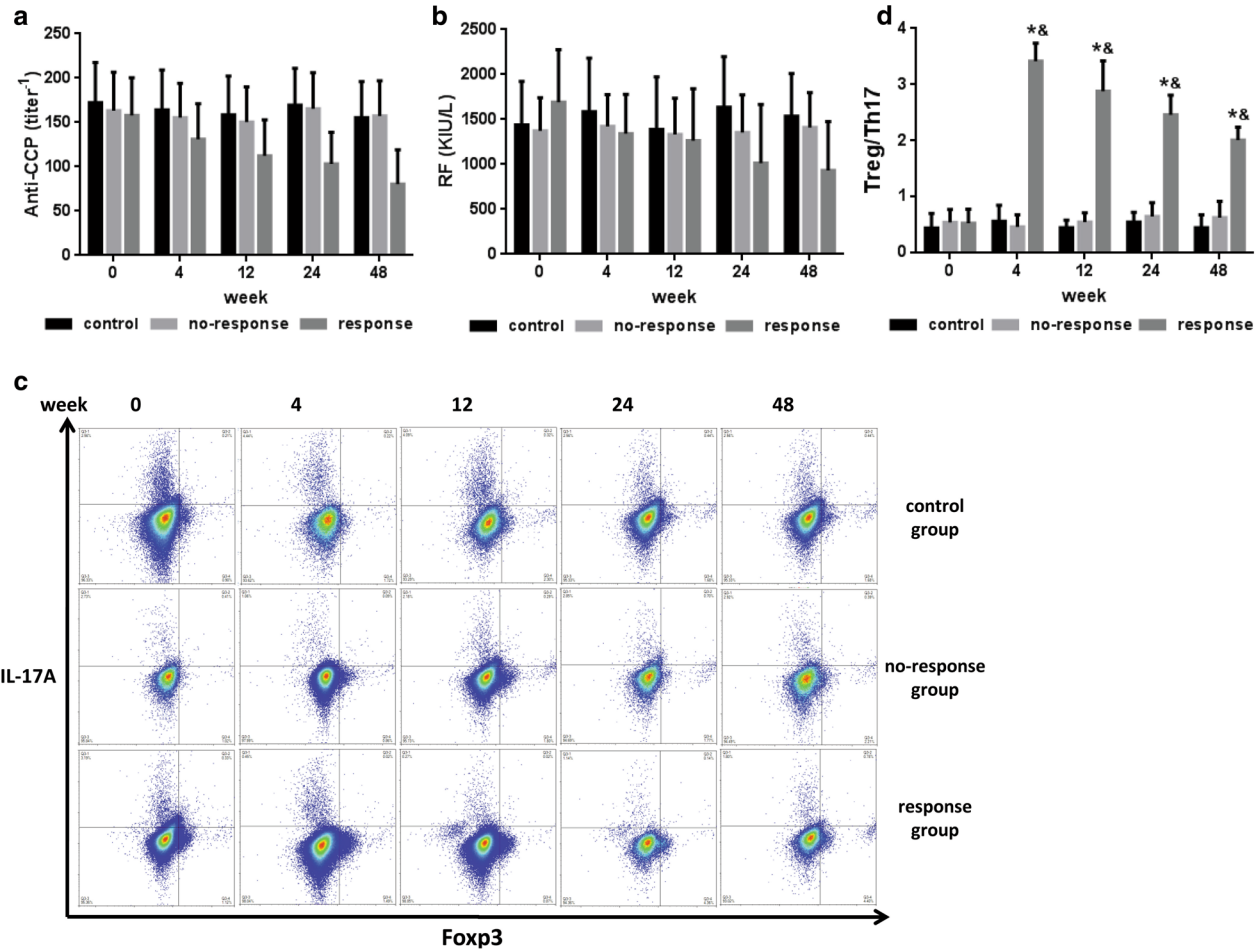
Serological autoantibody profiles and changes in immune function. Anti-CCP and RF levels and the ratio of CD4+Foxp3+ Tregs to CD4+IL-17A+ Th17 cells for CD4+ T cells were measured before and after MSCT for each group. **a** Serum anti-CCP level; **b** serum RF level; **c** flow cytometry results for Foxp3+ Tregs and IL-17A+ Th17 cells from CD4+ T cells for each group; **d** ratio of CD4+CD25+Foxp3+ Tregs to CD4+IL-17A+ Th17 cells for CD4+ T cells. *P < 0.05, versus the response group before MSCT; ^&^P < 0.05, versus the no-response and control groups according to one-way ANOVA. The error bars indicate the SEM. *Anti-CCP* anti-cyclic citrullinated peptide antibody, *RF* rheumatoid factor

To further investigate the therapeutic effects of MSCT, autoantibodies including anti-CCP and RF levels were detected before and after MSCT. Anti-CCP and RF levels were decreased after transplantation in the response group (Fig. 3a and b), however, these changes were not statistically significant.

### Immune function

RA is a severe, progressive, systemic inflammatory disease of unknown etiology. RA development is closely related to immune system disorders, which are mostly categorized by unbalanced immune homeostasis. Further tests showed that the percentage of CD4+CD25+Foxp3+ Tregs was increased and that the percentage of CD4+IL-17A+ Th17 cells was decreased in the response group compared to those in the no-response and control group (Fig. 3c). These findings support that MSCs play important roles in regulating immune homeostasis. In particular, a low ratio of CD4+CD25+Foxp3+ Treg to CD4+IL-17A+ Th17 cells (Treg/Th17) indicates an unbalanced immune status and excessive inflammation microenvironment. This low ratio was observed in all patients before MSCT, but it was gradually reversed in the response group after MSCT (Fig. 3d).

### Inflammatory microenvironment

**Fig. 4:**
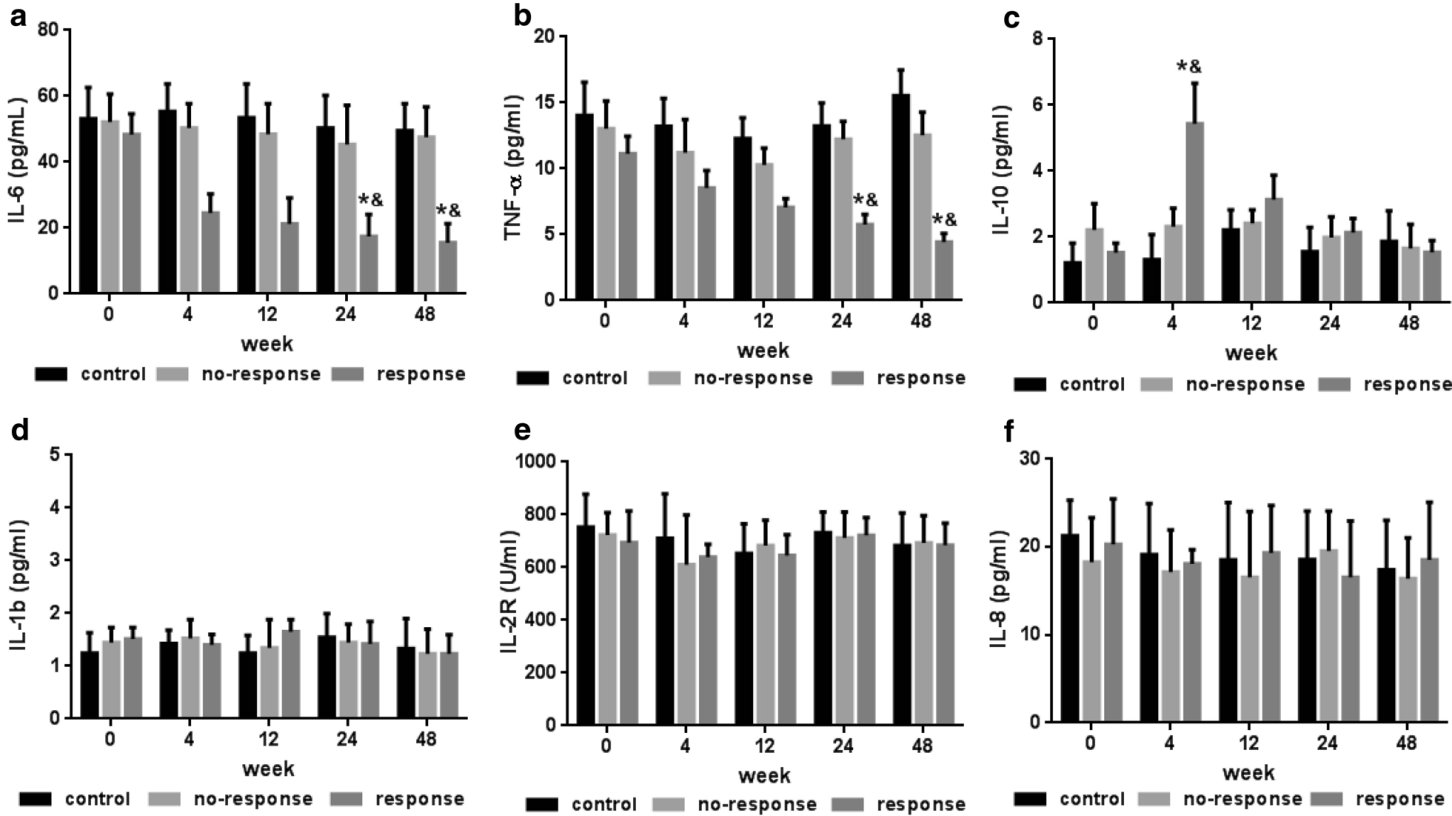
Inflammatory microenvironment measurements in RA patients before and after MSCT. **a** Serum interleukin 6 (IL-6) level; **b** serum tumor necrosis factor-alpha (TNF-α) level; **c** serum interleukin 10 (IL-10) level; **d** serum interleukin 1b (IL-1b) level; **e** serum interleukin 2 receptor (IL-2R) level; **f** serum interleukin 8 (IL-8) level. *P < 0.05, versus response group before MSCT; ^&^P < 0.05, versus the no-response and control groups according to one-way ANOVA. The error bars indicate the SEM

Because of their unbalanced immune status, RA patients are often characterized by a pro-inflammatory microenvironment, including high levels of interleukin (IL)-6 and tumor necrosis factor-alpha (TNF-α) (Fig. 4a, b) and low levels of IL-10 (Fig. 4c). After MSCT, patients in the response group showed improved immune balance, as manifested by decreased IL-6 and TNF-α levels (Fig. 4a, b) and increased in IL-10 levels (Fig. 4c). The IL-6 and TNF-α levels gradually decreased, whereas IL-10 levels increased significantly at 4 weeks and then gradually decreased to normal levels after MSCT in the response group. However, no significant changes in IL-1β, IL-2R and IL-8 levels were observed (Fig. 4d–f).

### IFN-γ predicts clinical response to MSCT in RA patients

**Fig. 5:**
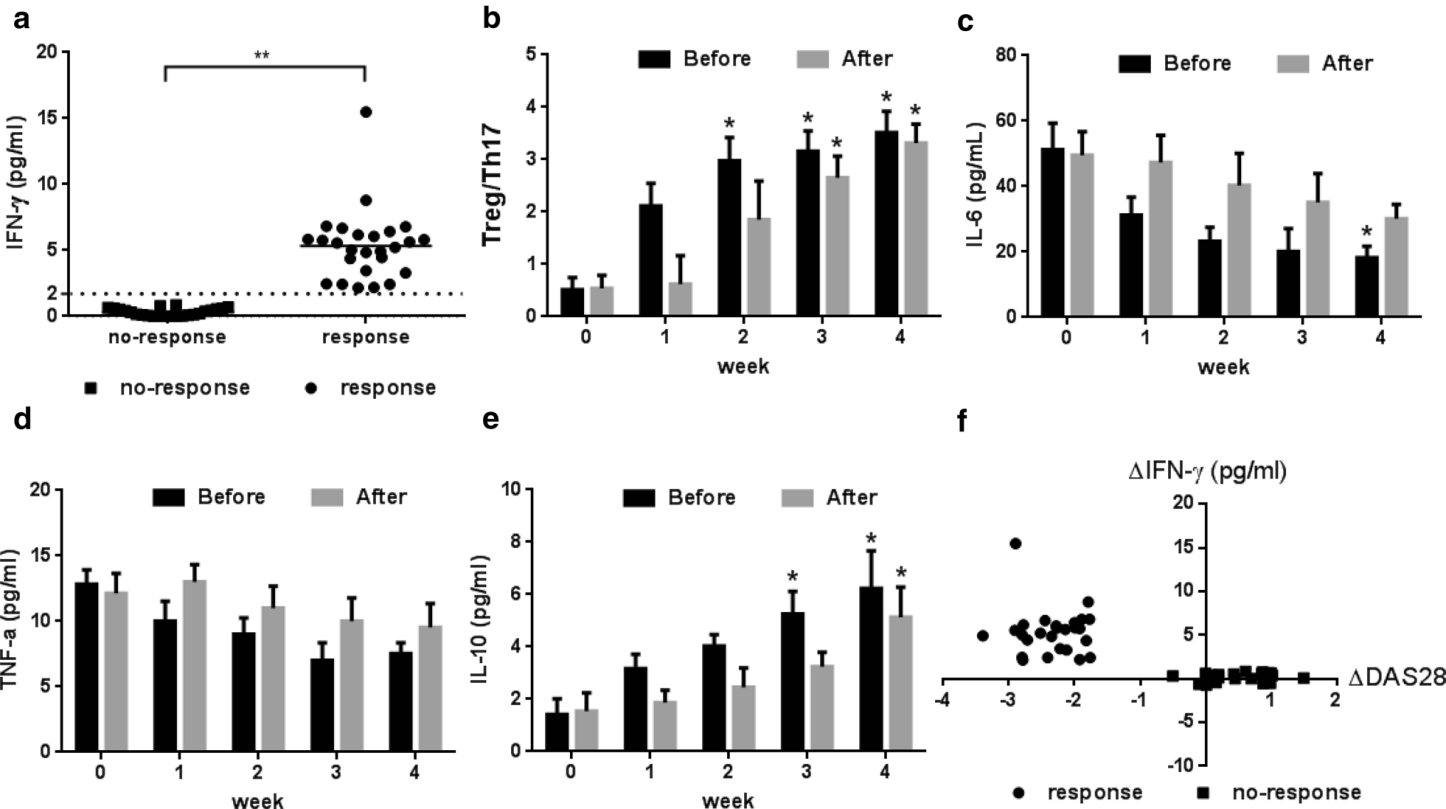
High endogenous IFN-γ levels were associated with a reduced DAS28 value in RA patients. **a** Peak IFN-γ level before and 4 weeks after MSCT for the response and no-response groups; **b** ratio of CD4+CD25+Foxp3+ Tregs to CD4+IL-17A+ Th17 cells levels for CD4+ T cells for the Before and After subgroups of the response group; **c** serum interleukin 6 (IL-6) levels for the Before and After subgroups of the response group; **d** serum tumor necrosis factor-alpha (TNF-α) levels for the Before and After subgroups of the response group; **e** serum interleukin 10 (IL-10) levels for the Before and After subgroups of the response group; **f** correlation between ∆DAS28 [DAS28 (12 weeks after MSCT) − DAS28 (before MSCT)] and ∆IFN-γ (IFN-γ peak before and 12 weeks after MSCT) for the response and no-response groups. *P < 0.05, versus the previous time. The error bars indicate the SEM. Before: subgroup of the response group with increased IFN-γ before MSCT (n = 12); After: subgroup of the response group with increased IFN-γ after MSCT (n = 16)

Allogeneic MSCT resulted in a clinical response rate of 54% (28/52) in our single clinical studies. Based on the aforementioned data, we sought to determine the reason why the same MSCs led to different outcomes for responders and non-responders enrolled from a single-center. It is well known that inflammation is an important target for treating autoimmune diseases, and the immunosuppressive properties of endogenous MSCs depend on the presence of IFN-γ in the microenvironment [12]. Besides, our in vitro study found that stimulated with IFN-γ could significantly increase the expression of indoleamine 2,3-dioxygenase (IDO), a key enzyme involved in the immunomodulatory ability of MSCs (Additional file 3: Figure S1). Therefore, we hypothesized that IFN-γ levels may play a key role in the different outcomes of the response group and no-response group after MSCT. As expected, high serum IFN-γ levels (>2 pg/mL) were generally observed before and 4 weeks after MSCT in the response group (Fig. 5a). This increase in IFN-γ levels was unrelated to infections since none of the patients had infectious complications. Importantly, IFN-γ levels remained low in the no-response group (Fig. 5a). The response group of patients was divided into Before and After subgroups according to whether a high serum IFN-γ level was observed before or after MSCT. Rapid therapeutic efficacy, according to the Treg/Th17 ratio, was found in the Before subgroup, and significant differences were observed 2–3 weeks after the IFN-γ increase (Fig. 5b). IL-6, TNF-α and IL-10 levels followed the same trend as the Treg/Th17 ratio, although some changes were not significant (Fig. 5c–e). Further studies revealed that the decrease in the DAS28 value (∆DAS28) at the 12-week evaluation was closely related to the increase in IFN-γ levels (∆IFN-γ) over the 12 weeks (Fig. 5f).

## Discussion

A decrease in the number of bone marrow MSCs can be found in RA-like autoimmune diseases, and this decrease grows more severe with disease progression [13]. Recent studies have shown that MSCs can ameliorate arthritis in RA patients and animal models and that the immunosuppressive effect of MSCs may be turned off or even switched to a stimulatory effect [14]. However, many aspects of the role of MSCs in treating RA remain unclear. Our present study has substantiated the clinical safety and efficacy of MSCT for treating RA patients. However, consistent with the study design, the clinical efficacy of MSCT varied greatly. Twelve weeks after MSCT, 54% of the patients in the MSCT group achieved a good or moderate response, whereas the other 46% of patients in the MSCT group had no clinical response.

The intravenous infusion of UCMSCs is a safe and effective practice that can improve immune function and serologic indices. In addition to a significant decrease in disease activity as assessed by the DAS28 value and HAQ score, MSC infusion reversed the inflammatory microenvironment and rebalanced the immune system. In the MSCT response group, evidence of the clinical benefits was obtained, and the clinical manifestation improvements were likely related to the increased Treg/Th17 ratio, which resulted in decreased levels of inflammatory cytokine expression, ESR and CRP. These results suggest that anti-inflammation along with improved immune-modulation and induced immune-tolerance are likely to be the major mechanisms of MSCT. However, the effects of MSCs in vivo are not permanent. In the present study, 8% of patients had disease relapse at 24 weeks after a prior good or moderate response. Disease activity indices, such as ESR and CRP levels, reverted slightly toward baseline levels, concomitant with relapsed joint swelling and pain. Because of the safety profile of MSC infusion in clinical applications, another MSC infusion after 6 months may be necessary for RA patients with partial relapse.

Considering there are no significant differences in age, gender and disease status between response and non-response groups, as mentioned in Additional file 1: Table S1, endogenous factors which may affect the immunoregulatory ability of MSCs come into our sight. To further investigate the cause of such varying responses, we focused on MSC responsiveness to the host microenvironment and MSC participation in immune homeostasis. Several reports have indicated that MSCs are not constitutively immunosuppressive; instead, they need to be activated by the inflammatory environment of the host to develop their immunoregulatory ability [15]. This finding was based on the observation that anti-IFN-γ receptor antibodies blocked the immunosuppressive effect of MSCs. In addition, the presence of other inflammatory cytokines can influence the phenotype and immunosuppressive effect of MSCs. Inflammatory stimuli induced-MSCs can secrete high levels of soluble molecules that can regulate immune homeostasis; these molecules include IDO, nitric oxide (NO), prostaglandin E2 (PGE2), TNF-α-stimulated gene 6 protein (TSG-6), heme oxygenase-1 (HO-1), chemokine (C–C motif) ligand 2 (CCL2), IL-10 and galectin [16]. In our in vitro study, we also confirmed that stimulated with IFN-γ could significantly increase the expression of IDO. According to data from animal models of RA, MSCs are most effective when transplanted after the onset of an inflammatory response. In a mouse GVHD model, MSC transplantation on the same day as bone marrow transplantation (BMT) showed no protective effects [17], whereas MSC transplantation 3, 8, or 20 days after BMT significantly ameliorated GVHD progression [18]. In line with this observation, we also observed increased IFN-γ levels prior to decreases in the DAS28 value and increases in the Treg/Th17 ratio and IL-10 levels. These data suggest that inflammation occurred before the MSCs exerted their immunosuppressive effects.

Since MSCT efficacy varies greatly among patients, biomarkers that predict MSCT response are urgently needed. Dander et al. [19] identified two biomarkers for acute GVHD, TNF receptor (TNFR) I and IL 2 receptor alpha (IL-2Rα), that may explain the patient response. The levels of these two factors were consistently decreased in the responder patients, but they were increased before the MSC infusion. This observation agrees with other studies indicating that MSCs need an inflammatory environment to be activated before exerting their therapeutic effects [15]. In the present study, we provide evidence that high serum IFN-γ levels, before or after MSCT, are positively associated with a reduced DAS28 value in RA patients and may be used as a biomarker to predict the clinical efficacy of or select RA patients for allogeneic MSCT.

Further investigation of IFN-γ-primed MSCs in animal models of RA will be crucial for developing novel MSC-based therapies for RA patients who respond poorly to MSCT. The current patients are still being followed, and more patients are being recruited to further confirm the safety and efficacy of MSCT as a new treatment modality for RA.

## Conclusions

In the present study, we demonstrate that (1) allogenic MSC transplantation is safe, as no serious adverse events were observed during or after MSC transplantation; (2) MSCT was effective in some RA patients, as suggested by the reductions in the DAS28 value, HAQ score, CRP level, and ESR; (3) MSCT improved the autoantibody profile of patients who responded to the therapy; (4) the potential mechanisms of MSCT are likely to be the anti-inflammatory or immunomodulatory effects of MSCs, as well as restored immune balance and tolerance; (5) serum IFN-γ levels may be a key factor that predicts the therapeutic effects of MSCs on RA patients.

## Additional files

**Additional file 1: Table S1.** Clinical and demographic characteristics of the patients enrolled in the study.

**Additional file 2.** Source and preparation of MSCs.

**Additional file 3: Figure S1.** IFN-γ increases IDO expression of MSC in vitro.

## Abbreviations

MSCT: mesenchymal stem cell transplantation
RA: rheumatoid arthritis
DAS28: 28-joint Disease Activity Score
HAQ: Health Assessment Questionnaire
Treg: regulatory T cell
Th17: T helper 17
EULAR: European League against Rheumatism
DMARDs: disease-modifying antirheumatic drugs
MSCs: mesenchymal stem cells
PBMCs: peripheral blood mononuclear cells
IFN-γ: interferon-γ
IDO: indoleamine 2,3-dioxygenase
GVHD: graft-versus-host disease
SLE: systemic lupus erythematosus
NSAIDs: non-steroidal anti-inflammatory drugs
UCMSCs: umbilical cord-derived human MSCs
anti-CCP: anti-cyclic citrullinated peptide antibody
RF: rheumatoid factor
ANOVA: one-way analysis of variance
CRP: C-reactive protein
ESR: erythrocyte sedimentation rate
IL-6: interleukin-6
TNF-α: tumor necrosis factor-alpha
TNFR: TNF receptor
IL-2Rα: IL 2 receptor alpha
