# Correction to: Serum IFN-γ levels predict the therapeutic effect of mesenchymal stem cell transplantation in active rheumatoid arthritis

**DOI:** 10.1186/s12967-021-02917-z

**Published:** 2021-09-23

**Authors:** Yi Yang, Xiao He, Rongseng Zhao, Wei Guo, Ming Zhu, Wei Xing, Dongpo Jiang, Chongyang Liu, Xiang Xu

**Affiliations:** 1 First Department, State Key Laboratory of Trauma, Burn and Combined Injury, Daping Hospital and Research Institute of Surgery, Third Military Medical University, Chongqing, 400042 People’s Republic of China; 2 Department of Rheumatology and Clinical Immunology, Daping Hospital and Research Institute of Surgery, Third Military Medical University, Chongqing, 400042 People’s Republic of China; 3 Department of Critical Care Medicine, Daping Hospital and Research Institute of Surgery, Third Military Medical University, Chongqing, 400042 People’s Republic of China

## Correction to: J Transl Med 16:165 (2018) https://doi.org/10.1186/s12967-018-1541-4

In the original publication [1] there was an error in Figure 3c. The 24 and 48 FACS plot were duplicated in Figure 3c control due to human error.

The updated 24 and 48 FACS plots are published in this correction article as Fig. 1 along with the original/incorrect figure (Fig. 2). The full captions are available via the original article. The original article has been updated.

**Fig. 1:**
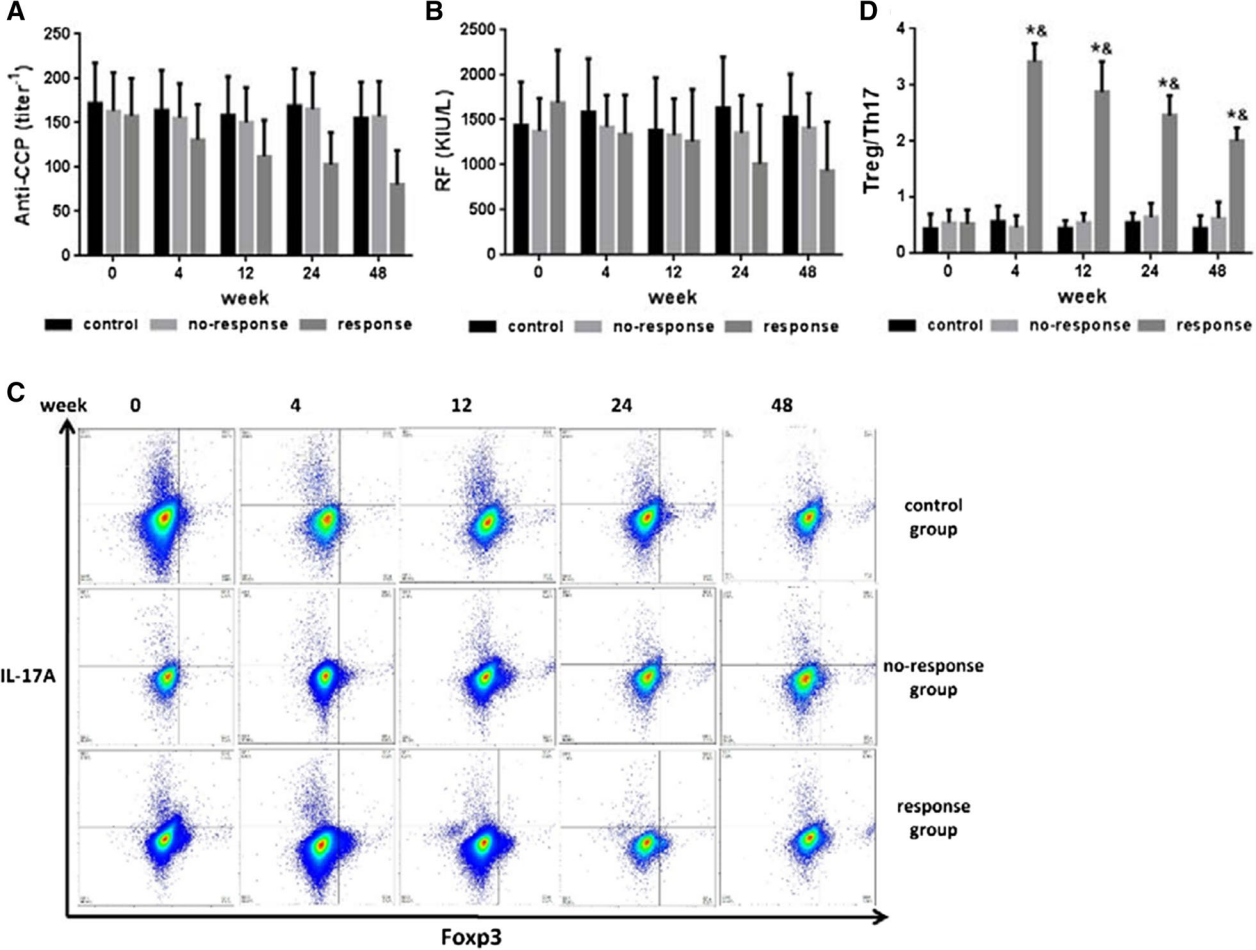
The corrected Figure 3c

**Fig. 2:**
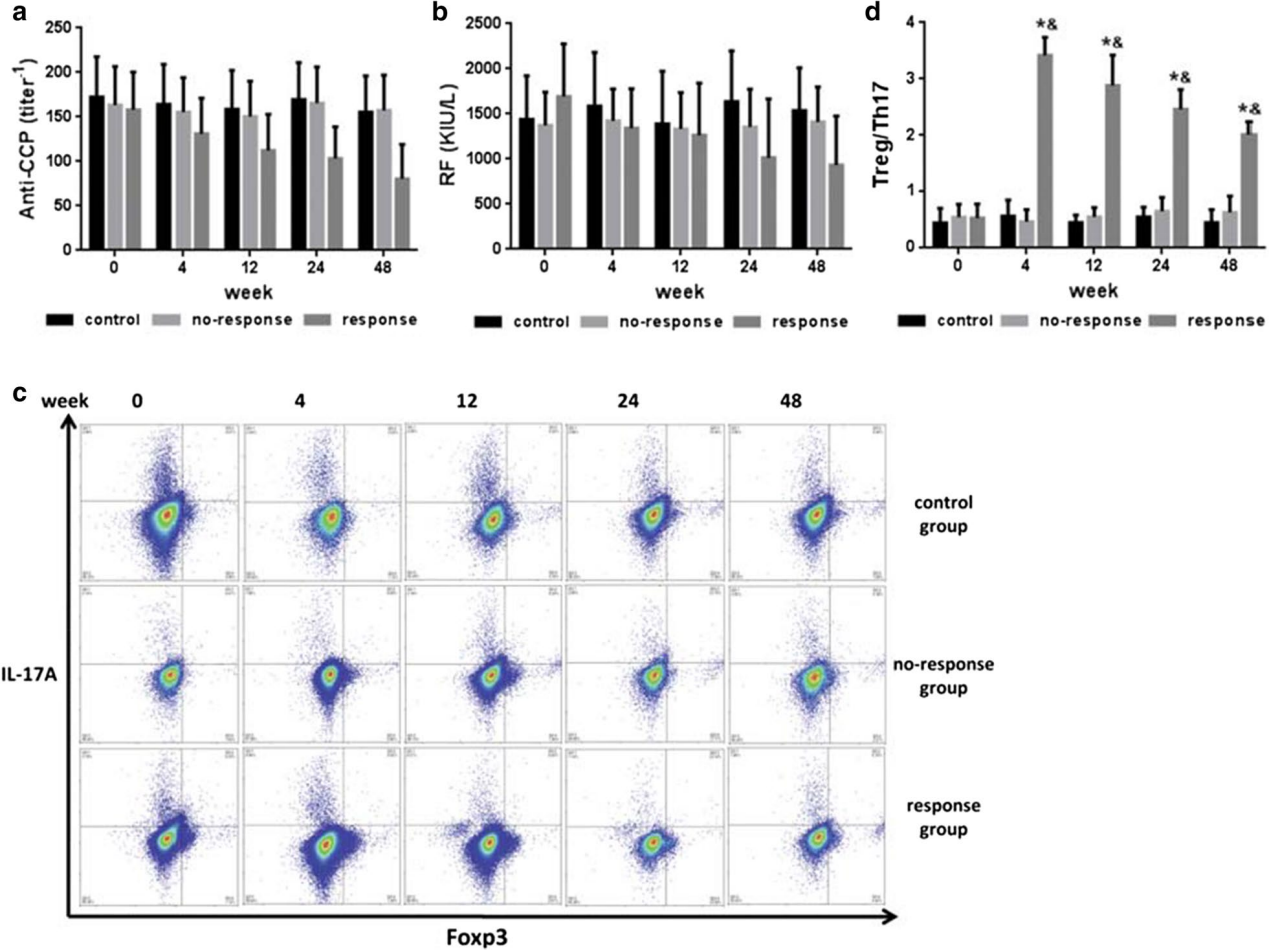
Incorrect Figure 3 as originally published

## References

[1] Yang Y, He X, Zhao R, Guo W, Zhu M, Xing W, Jiang D, Liu C, Xu X (2018). Serum IFN-γ levels predict the therapeutic effect of mesenchymal stem cell transplantation in active rheumatoid arthritis. J Transl Med.

