# Supplementary material for: Serum IFN-γ levels predict the therapeutic effect of mesenchymal stem cell transplantation in active rheumatoid arthritis
Source: J Transl Med. 2018 Jun 15;16:165. doi: 10.1186/s12967-018-1541-4 (PMC6003078; doi:10.1186/s12967-018-1541-4)
Supplement: Supplementary file 1 — Additional file 1: Table S1. Clinical and demographic characteristics of the patients enrolled in the study. [file 12967_2018_1541_MOESM1_ESM.doc]

**Table 1** Clinical and demographic characteristics of the patients enrolled in the study.

|  | MSCT group | | Control group |
| --- | --- | --- | --- |
|  | Response group | No-response group |  |
| Patients no. | 28 | 24 | 53 |
| Female no. (%) | 21 (75) | 19 (79) | 43(81) |
| Mean age (year) | 50.7 | 51.2 | 49.8 |
| Mean duration of disease (year) | 4±2.75 | 3.94±2.79 | 3.89±2.52 |
| DAS28 | 5.45±0.54 | 5.9±0.79 | 5.63±0.87 |
| HAQ | 1.62±0.17 | 1.68±0.23 | 1.59±0.21 |
| Medication history no. (%) |  |  |  |
| DMARDs | 28 (100) | 24 (100) | 53(100) |
| Biologics | 10 (35.7) | 10 (41.7) | 17 (32.1) |
| NSAIDs | 28 (100) | 24 (100) | 53(100) |
| Prednisone Acetate | 23 (82.1) | 22 (91.7) | 45(84.9) |

HAQ, the Health Assessment Questionnaire; DAS 28, the 28-joint disease activity score; DMARDs, disease-modifying anti-rheumatic drugs; NSAIDs, nonsteroidal anti-inflammatory drugs. Value: Mean±SEM.
