# Supplementary material for: Serum IFN-γ levels predict the therapeutic effect of mesenchymal stem cell transplantation in active rheumatoid arthritis
Source: J Transl Med. 2018 Jun 15;16:165. doi: 10.1186/s12967-018-1541-4 (PMC6003078; doi:10.1186/s12967-018-1541-4)
Supplement: Supplementary file 2 — Additional file 2. Source and preparation of MSCs. [file 12967_2018_1541_MOESM2_ESM.docx]

**Source and preparation of MSCs**

The allogeneic freshly cultured umbilical cord-derived human MSCs were all prepared from umbilical cord obtained from healthy full-term women aged 23, who underwent a physical examination and laboratory testing, including infectious disease testing, such as routine blood test, liver function and renal function tests, urinalysis and routine stool examination, T-spot test for tuberculosis, toxoplasma test, treponema pallidum antibody test, mycoplasma and chlamydia test, HIV test, and virus test including hepatitis virus, herpes simplex virus, cytomegalovirus, rubella virus, Epstein-Barr virus and human papillomavirus, and no abnormalities were found. A 10-15 cm fresh cord was obtained immediately after caesarean section at the Gynaecology Department of Third Affiliated Hospital, Third Military Medical University, after approval was obtained from the Institutional Ethics Committee. The samples were transferred to a class 10 000 cGMP-compliant clean room facility located at Daping Hospital of Third Military Medical University. After vessel removal, every 3-5 cm cord was cut into pieces (1-3 mm in diameter) with scissors and cultured in UltraCULTURE (Lonza, Walkersville, MD USA) (ULtroser G (Life Sciences, Port Washington, NY, USA), 2 mM L-glutamine) in a 25 cm^2^ cell culture flask (Corning, China). The adherent cells were detached enzymatically, re-plated into a cell culture flask and incubated at 37°C in a humidified atmosphere containing 5% CO_2_ to yield 500 to 900 million MSCs, which were cryopreserved at 5×10^6^ cells/mL to generate a master cell bank (MCB). These MSCs were limited to no more than 5 population doublings, had greater than 90% viability, were characterized according to spindle-shaped morphology, were positive (>95% expression) for CD29, CD44, CD90 and CD105 and negative (<5% expression) for CD34, CD45, and HLA-DR and were able to differentiate into adipogenic, osteogenic and chondrogenic lineages. The MSCs also tested negative for sterility, endotoxin, chlamydia and mycoplasma. We have proposed that non-cryopreserved, actively growing mesenchymal stromal cells would provide the best therapeutic effect unhindered by cryo-injury [1]. Therefore, the MSCs for use in the phase I trial were thawed from the MCB and propagated in culture (for a period of 5 to 10 days) on a weekly basis to constitute a single batch. These cells were collected enzymatically, washed, counted and prepared for patient infusion. Patients received 1×10^6^ cells/kg of body weight in 50 mL of 1% albumin in physiological saline as treatment. The final MSC product underwent an assessment of morphology and growth, showing greater than 90% viability with Trypan Blue staining and sterility through an endotoxin measurement of less than 2 EU. Based on Trypan Blue staining, the viability of the final cell product for the 15 MSC doses administered ranged from 90-95% (mean 93%).

**References**

[1] Galipeau J. The mesenchymal stromal cells dilemma--does a negative phase III trial of random donor mesenchymal stromal cells in steroid-resistant graft-versus-host disease represent a death knell or a bump in the road? Cytotherapy. 2013;15:2-8.
