# Supplementary figures and images for: Serum IFN-γ levels predict the therapeutic effect of mesenchymal stem cell transplantation in active rheumatoid arthritis
Source: J Transl Med. 2018 Jun 15;16:165. doi: 10.1186/s12967-018-1541-4 (PMC6003078; doi:10.1186/s12967-018-1541-4)

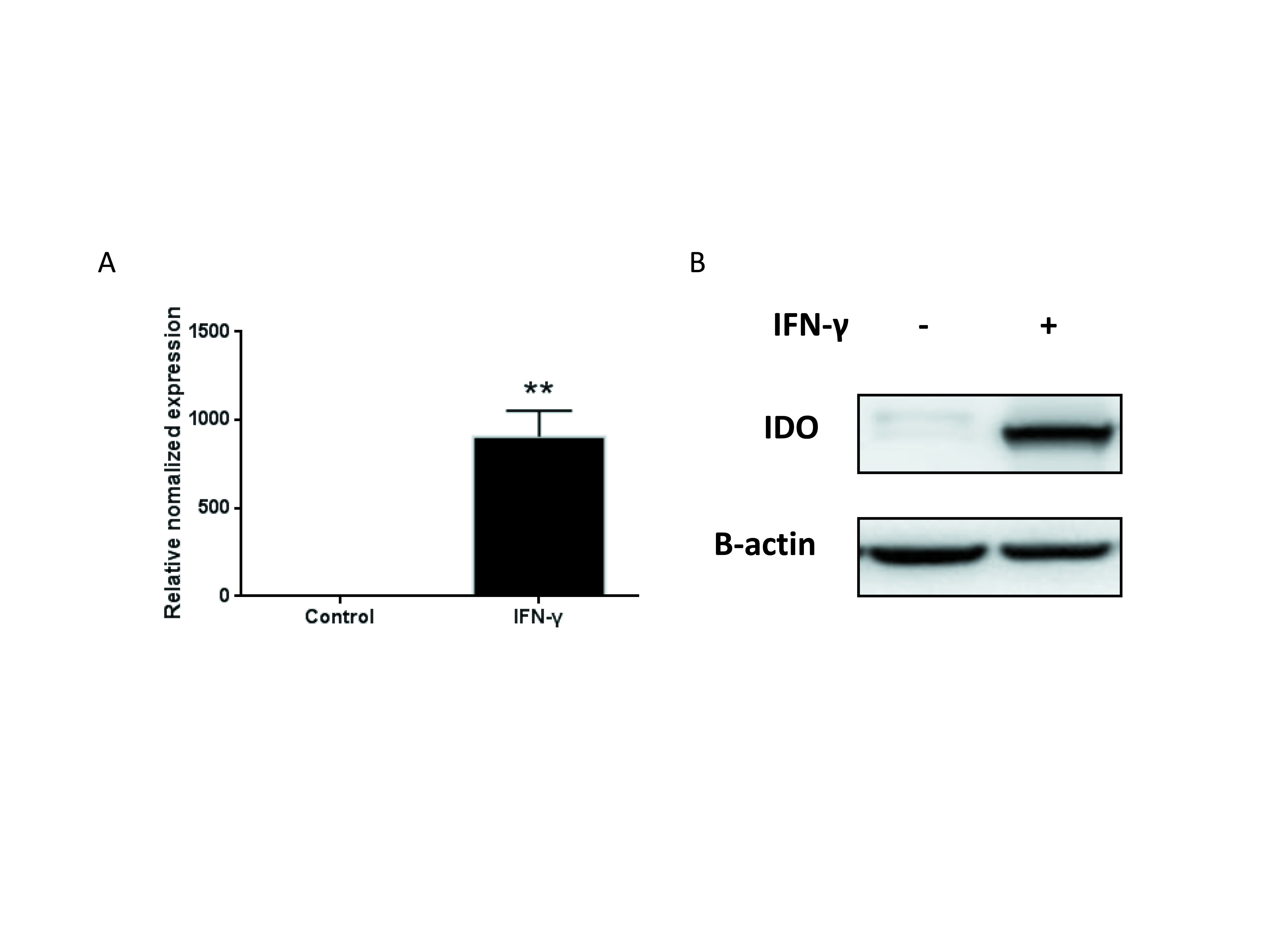

Supplement: Supplementary file 3 — Additional file 3: Figure S1. IFN-γ increases IDO expression of MSC in vitro. [file 12967_2018_1541_MOESM3_ESM.jpg]
